# STARR with Contour® Transtar™: prospective multicentre European study

**DOI:** 10.1111/j.1463-1318.2008.01714.x

**Published:** 2009-10

**Authors:** L Lenisa, O Schwandner, A Stuto, D Jayne, F Pigot, JJ Tuech, R Scherer, K Nugent, F Corbisier, E Espin-Basany, F H Hetzer

**Affiliations:** * Department of Surgery, Casa di Cura San Pio XMilan, Italy; † Department of Surgery, Caritas-Krankenhaus St. JosefRegensburg, Germany; ‡ Department of Surgery, Ospedale S. Maria degli AngeliPordenone, Italy; § Academic Surgical Unit, St James University HospitalLeeds, UK; ¶ Department of Proctology, Bagatelle-Maison de SanteTalence-Cedex, France; ** Department of Digestive Surgery, University HospitalRouen, France; †† Department of Surgery, Krankenhaus WaldfriedeBerlin, Germany; ‡‡ Department of Surgery, General HospitalSouthampton, UK; §§ Department of Surgery, C.H.N.D.R.F.Charleroi, Belgium; ¶¶ Department of Surgery, Hospital Valle de HebronBarcelona, Spain; *** Department of Surgery, Cantonal HospitalSt Gallen, Switzerland

**Keywords:** STARR, obstructive defecation syndrome, constipation, internal rectal prolapse, rectocele, incontinence

## Abstract

**Objective:**

The stapled transanal rectal resection (STARR) in patients with defecation disorders is limited by the shape and capacity of the circular stapler. A new device has been recently developed, the Contour® Transtar™ stapler, in order to improve the safety and effectiveness of the STARR technique. The study has been designed to confirm this declaration.

**Method:**

From January to June 2007 a prospective European multicentre study of consecutive patients with defecation disorder caused by internal rectal prolapse underwent the new STARR technique. The assessment of perioperative morbidity and functional outcome after 6 weeks, 3 and 12 months was documented by different scores.

**Results:**

In all 75 patients, median age 64, the Transtar procedure was performed with 9% intraoperative difficulties, 7% postoperative complications and no mortality. The mean reduction of the ODS score was −15.6 (95%−CI: −17.3 to −13.8, *P* < 0.0001), mean reduction of SSS was −12.6 (95%−CI: −14.2 to −11.2; *P* < 0.0001). 41% stated improvement of their continence status by CCF score, only 4 patients (5%) had deterioration.

**Conclusion:**

The Transtar procedure is technically demanding, with good functional results similar to the conventional STARR.

## Introduction

Ano-rectal intussusception, observed in patients with outlet obstruction, rather than any associated rectocele, has been claimed to be a major determinant of difficult evacuation [1]. This may explain why previous surgery aimed at correcting the rectocele frequently failed to control obstructive defecation syndrome (ODS) [2]. The observation of improved evacuation after stapled rectopexy suggested a role for internal rectal prolapse and rectal intussusception, with or without rectocele, in ODS. This led to the development of Stapled Trans-Anal Rectal Resection (STARR) [3], which aimed to remove redundant rectum and to restore normal rectal anatomy. Encouraging results were reported by many authors [4–10], but the commonly used prolapsing haemorrhoidal stapler, PPH 01 (Ethicon Endo-Surgery; Cincinnati, OH, USA), has limitations in the amount of rectal wall that can be resected; furthermore, the use of a circular stapler also requires retraction of the opposite rectal wall with a retractor. In addition, resection is performed ‘blind’ after trans-anal insertion of the stapler. These technical limitations may explain some of the difficulties and complications experienced with the STARR technique [11–14].

A new device has been designed to overcome these difficulties. The Contour® Transtar™ stapler (Ethicon Endo-Surgery; Cincinnati) is designed to allow tailored modulation of the amount of rectal wall to be resected and to improve open visualization of the procedure.

This study has been designed by a multi-national European group of surgeons experienced in the conventional double circular stapler STARR procedure. Its aim was to assess the feasibility of the Contour® Transtar™ stapler and to record functional results up to 1 year.

## Method

### Study design

A prospective multicentre trial was designed in patients with internal rectal prolapse or intussusception with or without rectocele. Outcome parameters included perioperative morbidity and postoperative functional outcome. The intra-operative data analysed included operative time, complications and technical aspects, such as the number of used cartridges and weight of the resected specimen. Hospital stay and postoperative complications, graded in accordance with the severity score of Dindo *et al.* [15], were documented. The severity of the functional defecation disorder was assessed in every patient before and after surgery by the ODS score [16], severity of symptoms score (SSS) [17] and the Jorge-Wexner continence score (CCF) [18]. The follow-up outpatient visits were scheduled at 6 weeks and 3 and 12 months.

Eleven centres from seven European countries contributed patients to the trial. All investigators were experienced in the STARR technique with the double PPH 01 [3]. They were required to complete a 2-day training programme at the education centre of Ethicon EndoSurgery (Norderstedt, Germany). Immediately after the training, all STARR procedures using the new device in every centre were monitored by the preceptor AS (Co-author). The procedure, Stapled Trans-anal Rectal Resection (STARR) with Contour® Transtar™ Curved Cutter Stapler, from hereon is referred to as ‘Transtar’.

### Patient selection

From January to June 2007, consecutive patients with defecation disorder caused by rectal redundancy were eligible for enrolment. Conservative treatment with diet, laxatives, enemas and/or physiotherapy had been tried in all patients without success. Rectal redundancy, intussusception with or without anterior rectocele, were diagnosed by clinical examination and confirmed by dynamic magnetic resonance (MR)- or conventional defecography. Exclusion criteria for a Transtar procedure were in accordance with the consensus statement recently published by the Pioneers group [19]. These included patients with concurrent severe ano-rectal pathology (including anal stenosis), active ano-rectal infection, proctitis, chronic diarrhoea and previous anterior resection with rectal anastomosis, as well as patients with any foreign material (such as mesh) adjacent to the rectum or with a psychiatric disorder. Patients with a low fixed enterocele at rest, external rectal prolapse and paradoxical contraction of the puborectalis and sphincter muscles (anismus) diagnosed by proctography and manometry, were also not considered straightforward candidates for a feasibility study. Preoperatively, all patients were evaluated by a full history, physical examination and laboratory tests according to local clinical guidelines.

### Surgical technique

**Figure 1:**
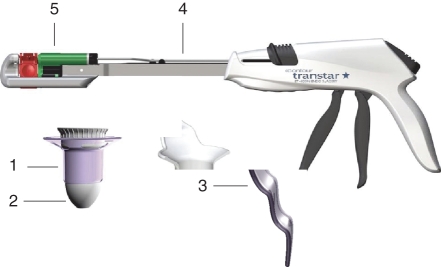
The Contour® Transtar™ curved cutter-stapler kit includes: (1) circular anal dilatator (CAD), (2) obturator, (3) access suture anoscope (ASA), (4) contour transtar™ stapler and (5) contour transtar cartridge reload (CR30G).

Preoperative preparation included one or two phosphate enemas the morning of surgery, routine deep vein thrombosis prophylaxis and perioperative broad spectrum antibiotics. General or regional anaesthesia was used based on the individual surgeon’s preference. The patient was placed in the lithotomy position with the hips in hyperflexion. An initial examination was undertaken to confirm the presence and extent of the internal rectal prolapse and rectocele and also to confirm the absence of co-existent pathology. The Contour® Transtar™-STR5G (Ethicon EndoSurgery Inc., Cincinnati, OH, USA) stapling kit was opened (Fig. 1) and the circular anal dilator (CAD) gently introduced and fixed to the perianal skin with four cardinal 1/0 silk sutures. A swab was inserted and gently pulled outward to visualize the apex of the intussusception.

#### Step 1: parachute suture placement

**Figure 2:**
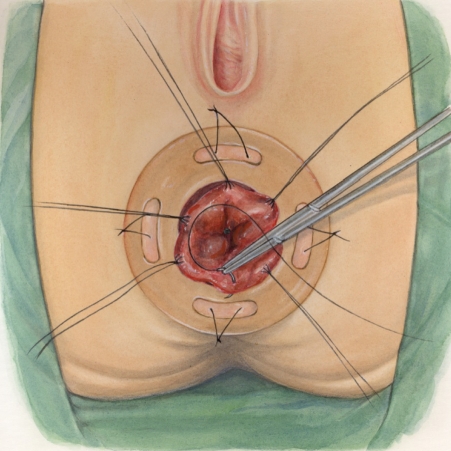
The ‘Parachute Suture’: 4–6 stitches are placed superficially on the apex of the intussusceptions to obtain a uniform circumferential traction.

An initial 2/0 prolene traction suture was placed at the 2 o’clock position into the apex of the intussusception and two or three further full-thickness bites were taken so that the needle exited at the 1 o’clock position when the suture was loosely tied. Working anticlockwise, similar sutures were placed between the 12 and 11 o’clock, 10 and 9 o’clock, 8 and 7 o’clock, 6 and 5 o’clock and 4 and 3 o’clock positions, resulting in six traction sutures placed circumferentially around the apex of the intussusception, leaving a gap between 4 and 2 o’clock for the opening radial staple cut (Fig. 2).

#### Step 2: opening of the prolapse

**Figure 3:**
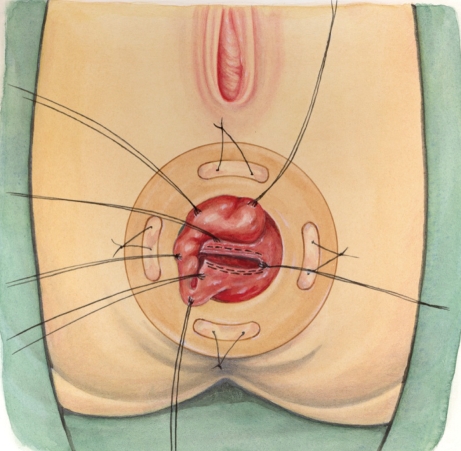
The prolapsed has been open in longitudinal direction with the stapler device at 3 o’clock. An orientation suture is placed at the end of the opened intussusception, thus marking the ending of the following circumferential resection and reinforcing the anastomosis.

A 5th traction suture was placed at the 3 o’clock position at the point of opening of the prolapse, and this was tied tightly such as to be able to collapse the tissue like a concertina. A loop was made in the end of this suture through which the Transtar stapler was passed into the distal rectum. Traction was applied to the 3 o’clock suture to bring the prolapse into the jaws of the stapler, and the stapler retaining pin was then inserted and the stapler was closed. A period of 15 s was allowed between closing and firing of the stapler to maximize tissue compression and subsequent haemostasis, during which time a vaginal examination was performed to ensure that none of the posterior vaginal wall had been included. The stapler was fired resulting in a radial cut into the prolapse, opening up the intussusception. A Vicryl marker suture (20) was placed at the apex of the radial cut to act as a reference point for the beginning and end of the circumferential resection and to prevent ‘spiralling’ of the staple line. One thread of the loop was pulled into the head of the device and the other pulled behind. During the first cut, the head of the device was held radial to the CAD, to open the intussusception. The retaining pin was closed manually; checking the vagina with a finger and the device was closed and fired. It was then removed from the rectum. An orientation suture was placed at the end of the opened intussusception, thus marking the end of the circumferential resection (see below) reinforcing the anastomosis (Fig. 3).

#### Step 3: circumferential resection

**Figure 4:**
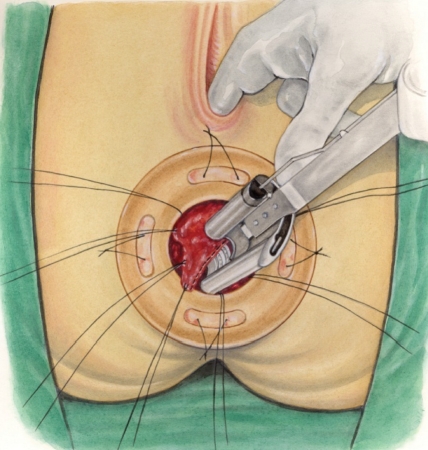
The device, Contour® Transtar™ Curved Cutter Stapler, is introduced in the rectum, placed parallel to the circular anal dilatator and moved counter clockwise. The vagina is checked with the finger; the device is then closed and fired. The cartridge will be replaced and the operation is repeated all along the circumference.

After replacing the stapler cartridge, the device was re-introduced into the rectum and rotated anticlockwise with traction on the 2 to 12 o’clock and 11 to 9 o’clock sutures to bring the redundant anterior prolapse into the jaws of the stapler. The retaining pin and the stapler were closed and the vagina checked prior to firing the stapler (Fig. 4). The resection proceeded in a anticlockwise direction until a full-thickness circumferential resection had been performed. Particular care was taken with the final stapler firing, using the marking suture at the 3 o’clock position as a reference point, to ensure that the resection terminated at the same position as it had begun. The final staple line was inspected for bleeding which were secured with interrupted 3/0 Vicryl as required. Reinforcement sutures were placed as deemed necessary, but particularly at the intersection of individual staple lines. The resection specimen was sent for histological analysis which included the height and weight of the specimen, evidence of the presence of full-thickness rectal wall, and the presence/absence of peritoneum.

### Postoperative course

The patients were started on a low-fibre and easily digestible diet from the first postoperative day. No further antibiotics were administered. The patients were given analgesics including non steroidal anti-inflammatory agents and morphine as required. Low molecular heparin was given until discharge from hospital, which depended on the patient’s pace of recovery. Patients were closely assessed for any complication.

Monitoring of complications and adverse events was followed up in the outpatient clinic at 6 weeks, 3 and 12 months postoperatively. Data were collected on a web-based database with each participating centre having an exclusive ID and password.

### Statistics

All data were coded and analysed using spss 13 (Statistical Package for the Social Sciences; SPSS, Chicago, IL, USA). Comparisons between preoperative and postoperative scores were performed using the nonparametric Mann–Whitney *U*-test, Kruskal–Wallis test where appropriate. Qualitative data were compared using chi-square test or two tailed Fisher exact test. Values of *P*<0.05 were considered statistically significant.

## Results

### Demographics, surgical history and preoperative findings

During the period between January 2007 and June 30, 2007, 75 patients having trans-anal rectal resection using the Contour® Transtar™ were entered into the prospective registry. All had been followed up for 12 months. The majority were females (97%) with a median age of 64 (range 20–83) years and a median body mass index (BMI) of 25 (range 17–40) kg/m^2^. Thirty-two per cent had a previous hysterectomy, 16% an urogynaecological procedure prior to STARR, and 19% had minor ano-rectal surgery. An anterior rectocele was present in 93% and 76% and 51% had an internal rectal prolapse and/or rectal mucosal prolapse. Perineal descent was present in 49%, and 8% of patients had a concomitant enterocele.

### Safety of the procedure

The median operative time was 45 (ranging from 24 to 90) min and the median hospitalization was 4 (ranging from 1 to 16) days. The median width and length of the resected specimen were 81 and 48 mm respectively, and the median weight was 30 g. A median number of six (ranging from four to nine) cartridges was used. Histology of the specimen showed full-thickness rectal wall with perirectal fat in all cases. Postoperatively, the first bowel movement occurred after a median of 2 (ranging from 1 to 4) days.

Seven (9%) intra-operative difficulties were reported. These included partial dehiscence of the staple line in four patients requiring immediate additional suturing (with no further surgical re-intervention), and spiral resection in three patients requiring conservative treatment by observation and oral antibiotic medication, but no further surgical treatment. Postoperatively, five (7%) complications occurred including two grade IIIb (bleeding in two patients requiring re-operation), two grade IIIa (bleeding requiring rectoscopic haemostasis and one urinary retention with the need for catheterization), and one grade II (hypotensive episode treated medically). There was no death or serious morbidity such as rectovaginal fistula formations.

### Efficacy of the procedure

**Figure 5:**
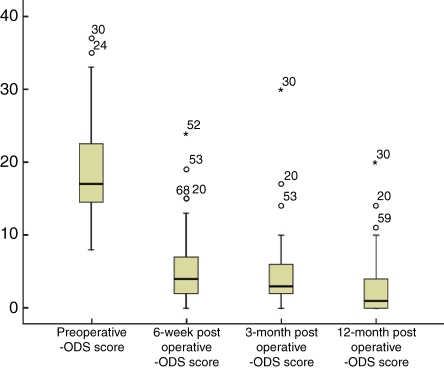
Obstructive defecation syndrome (ODS) score before, 6 weeks, 3 and 12 months after Transtar procedure. Median values, 25th to 75th percentiles and fifth to 95th percentiles are denoted by horizontal bars, boxes and error bars respectively.

**Figure 6:**
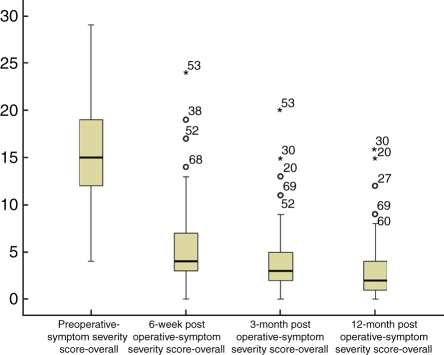
Severity of symptoms score before, 6 weeks, 3 and 12 months after Transtar procedure. Median values, 25th to 75th percentiles and fifth to 95th percentiles are denoted by horizontal bars, boxes and error bars.

A statistically significant reduction in both ODS and SSS scores was observed at a 12-month follow-up (Tables 1 and 2). Overall, 77.3% (*n* = 58) of the patients experienced improvement of ODS and 22.7% had no change. The changes in ODS and SSS are shown in Figs 5 and 6. The mean reduction of the ODS was −15.6 (95% CI: −17.3 to −13.8; *P*<0.0001), and of the SSS −12.6 (95% CI: −14.2 to −11.2; *P*<0.0001).

**Table 1:** The obstructed defecation syndrome score before and at 12 months after the Transtar™ stapling procedure.

ODS symptoms	Preoperative, mean (SD)	12 months, mean (SD)	Difference, mean
Defecation frequency	1.3 (1.04)	0.2 (0.42)	−1.0*
Intensive straining	1.4 (0.62)	0.4 (0.52)	−1.2*
Time spent on defecation	1.7 (0.47)	0.4 (0.59)	−1.3*
Incomplete defecation	2.5 (0.86)	0.6 (0.86)	−2.0*
Pain	1.7 (1.65)	0.0 (0.20)	−1.6*
Impact on daily routine	2.6 (1.85)	0.3 (0.89)	−2.5*
Laxatives	3.0 (2.54)	0.7 (1.55)	−2.5*
Use of enemas	1.2 (2.09)	0.5 (0.21)	−1.3*
Digital assistance	2.2 (2.68)	0.0 (0.00)	−2.4*
Total score	17.6 (7.02)	3.0 (3.89)*	−15.6*

* *P* < 0.0001.

**Table 2:** Severity of symptoms score before and at 12 months after Transtar™ stapling procedure.

Symptoms	Preoperative, mean (SD)	12 months, mean (SD)	Difference, Mean
Laxatives/enemas	3.0 (1.33)	1.6 (1.06)	−1.4*
Unsuccessful defecation	3.3 (1.26)	1.3 (0.58)	−2.1*
Decreased defecation frequency	2.6 (1.23)	1.4 (0.66)	−1.1*
Prolonged defecation/straining	3.6 (1.25)	1.5 (0.73)	−2.3*
Pain	2.7 (1.32)	1.1 (0.42)	−1.6*
Incomplete evacuation	3.7 (1.33)	1.4 (0.77)	−2.4*
Bleeding	2.0 (0.99)	1.0 (0.18)	−1.0*
Soiling	1.8 (1.17)	1.2 (0.38)	−0.7*
Difficulties to hold stool (urgency)	1.8 (1.13)	1.7 (1.14)	−0.1
Total score	24.5 (5.71)	12.2 (3.13)*	−12.6*

* *P* < 0.0001.

### Faecal incontinence and urgency

The mean preoperative CCF incontinence score (3.5) was reduced to 1.0 at 12 months giving a mean reduction of −2.7 (95% CI: −4.0 to −1.5, *P* < 0.0001). Forty-one per cent of patients stated improvement of their continence status by CCF score and four (5%) patients had a deterioration of continence. Faecal urgency assessed by the SSS score among 73 patients available for analysis occurred for the first time in 13% (*n* = 10) 12 months after trans STARR and pre-existing urgency resolved in 22% (*n* = 16) in whom it was present preoperatively.

## Discussion

Stapled Trans-Anal Rectal Resection has been proposed for the treatment of the ODS in the presence of internal rectal prolapse (rectal intussusception) and associated rectocele. This is the first report with medium-term results of this technique performed with the new Contour® Transtar™ Curved Cutter Stapler. The device has been developed to overcome the potential drawbacks of the original STARR using the haemorrhoidal stapler (PPH 01). This procedure is technically demanding and there is a learning curve period during which resection is difficult in about a tenth of cases. In the present trial, the procedure had a low postoperative morbidity (7%) and reduced obstructed defecation in all patients. More than 90% of our patients had an anterior rectocele while only 8% had an enterocele. Our main inclusion criterion was a full-thickness internal rectal prolapse associated with ODS.

The new device effectively allows a full-thickness resection of the entire rectal circumference. This was consistent with the mean size and weight of the specimens obtained, which were at least twice the weight of a standard double-stapler specimen. The length of rectal wall to be resected may be tailored to the patient’s anatomy and surgeon’s choice and is not limited by the device. It is not known whether resecting more tissue will improve the functional outcomes further but the technique with the Contour® Transtar™ instrument proved satisfactory in our hands. It was however found to be more demanding than the procedure with the conventional STARR PPH instrument. Dehiscence and spiralling of the staple line were detected intra-operatively in 9% of procedures, with the need for manual oversewing although this had no impact on the postoperative morbidity in any of the patients with obstructed defecation pathology.

Spiral resection may be a technical problem specifically related to this procedure. It is caused by the staple line ending inwards or outwards with respect to the beginning of the suture line; leaving an island of tissue in the gap between the two lines which may potentially cause anastomotic leakage. In the learning phase, this may have resulted from excessive or uneven traction on the parachute stitches at the edge of the prolapse or to excessive thickness of tissue incorporated into the jaws of the device. If this is so, the height and depth of the lateral stitch at 3 o’clock appears to be critical for determining the height of the staple line, as well as the amount of tissue to be resected. Immediate detection of possible staple-line leakage is crucial. The low postoperative complication rate of 7% contrasts with 16% (0 [10]–38% [11]) reported for the conventional STARR procedure with the PPH instrument. Other than two cases of bleeding from the stapler line, there were no complications which required re-operation with anaesthesia. There were no cases of pelvic floor sepsis [11], recto-vaginal fistula formation [20], rectal diverticulum [14,20] or persisting pelvic pain [13,20], all of which have been described following the STARR procedure. The Transtar appears to be as safe as the PPH-STARR and the complication rate reported in this study is acceptable. Furthermore, more extensive resection does not appear to be associated with a higher rate of complications or an increased safety of the device.

Incontinence has been claimed to be a potential postoperative drawback of STARR [12,21] and for some its presence may be a contraindication to trans-anal surgery for ODS [22]. Impaired continence may however be part of the symptom aetiology of patients with internal rectal prolapse and an intact sphincter and not by itself a contra-indication to surgery. In this study, only 5% of the patients complained of new onset incontinence, while 41% of these with a degree of incontinence preoperatively that were improved at 1 year after operation. Faecal urgency is commonly considered to be part of continence disorder and is not accounted for separately in the Cleveland continence system. It is however an independent item of the SSS, which showed a new onset of urgency in 13% of patients. It is noteworthy however that 22% of the patients who had urgency preoperatively were relieved of this symptom by the operation.

Patients should be carefully selected for the STARR procedure. The present authors have previously developed an algorithm for patient selection for the STARR procedure [19]. The strict adoption of the algorithm for the entry of patients into this study, combined with extensive experience with STARR using double PPH and training in the use of the new device may explain the results achieved in this study.

The Contour® Transtar™ device appears to facilitate more tailored surgery, including a real circumferential full-thickness resection with the potential of removing more tissue. This may lead to an improved functional outcome. Reports of function after the STARR with PPH have been encouraging [4–10] and the Transtar procedure seems to produce results at least as good as that judged by constipation scoring systems. This might suggest that larger resections may perhaps not be the sole factor determining a good postoperative outcome, but it is our opinion that STARR with Transtar is a more satisfactory technique for trans-anal rectal resection.
